# Identifying key genes related to the peritubular capillary rarefaction in renal interstitial fibrosis by bioinformatics

**DOI:** 10.1038/s41598-023-46934-y

**Published:** 2023-11-10

**Authors:** Yu Zhang, Chuanbing Shi, Yiqiong Yang, Xiuxiu Hu, Haifeng Ni, Li Li, Zhengyuan Cheng, Jing Huang, Pingsheng Chen

**Affiliations:** 1 Department of Pathology, School of Medicine, Southeast University, Nanjing, Jiangsu China; 2 Department of Pathology, Pukou Branch of Jiangsu People’s Hospital, Nanjing, Jiangsu China; 3 Institute of Nephrology, Zhong Da Hospital, School of Medicine, Southeast University, Nanjing, Jiangsu China; 4 Department of Internal Medicine, Ma’anshan People’s Hospital Affiliated to Medical School of Southeast University, Ma’anshan, Anhui China; 5 Department of Respiratory and Critical Care Medicine, Zhongda Hospital, School of Medicine, Southeast University, Nanjing, Jiangsu China

**Keywords:** Transcriptomics, Biomarkers, Renal fibrosis

## Abstract

Renal interstitial fibrosis (RIF) is a key feature of progressive chronic kidney disease (CKD), characterized by tubular epithelial cell (TEC) hypoxia and peritubular capillary (PTC) rarefaction. However, the mechanisms underlying these processes remain poorly understood. To address this knowledge gap, we conducted a comparative transcriptome analysis of hypoxic and normoxic HK-2 cells, identifying 572 differentially expressed genes (DEGs). Subsequent Gene Ontology (GO), protein‒protein interaction (PPI) network, and hub gene analyses revealed significant enrichment of DEGs in the HIF-1 signaling pathway based on KEGG enrichment analysis. To further explore TEC modulation under hypoxic conditions, we performed chromatin immunoprecipitation (ChIP) sequencing targeting HIF-1α, identifying 2915 genes potentially regulated by HIF-1α. By comparing RNA sequencing and ChIP sequencing data, we identified 43 overlapping DEGs. By performing GO analysis and peak annotation with IGV, we identified two candidate molecules, VEGFA and BTG1, that are associated with angiogenesis and whose gene sequences were reliably bound by HIF-1α. Our study elucidates the molecular mechanisms underlying RIF, providing valuable insights for potential therapeutic interventions.

## Introduction

Chronic kidney disease (CKD) is often referred to as an invisible epidemic, with a global prevalence of a staggering 9.1%, impacting approximately 697.5 million patients. In China alone, an estimated 132.3 million individuals suffer from CKD^1–3^. The rising incidence of diabetes and hypertension, which are well-known risk factors for CKD, has further exacerbated the global burden of this condition^4^.

Renal interstitial fibrosis (RIF), a critical hallmark and prevalent biological alteration in CKD, plays a significant role in the progression toward end-stage renal disease (ESRD) and is associated with notable peritubular capillary (PTC) rarefaction^5,6^. Previous studies have highlighted a notable phenomenon wherein destruction of the microvascular network (vessels of 20–200 μm in diameter) occurs more frequently than the loss of medium and large blood vessels during the progression of chronic RIF^7,8^. Importantly, the imbalance of capillary degeneration and neoformation around the renal tubules contributes to tissue hypoxia. Simultaneously, continuous exposure to a hypoxic microenvironment exacerbates pathologic sprouting angiogenesis, leading to significant activation and proliferation of myofibroblasts and deposition of extracellular matrix (ECM)^9^. Despite the conclusive findings in this area, the mechanisms underlying PTC deficiency in RIF remain poorly understood due to its complexity, which depends on the delicate regulation of proangiogenic and antiangiogenic factors^10^. Currently, promising therapeutic approaches for vascular normalization are limited. Therefore, there is a need to explore more sensitive biomarkers.

Multiple cell types have been reported to play a role in PTC loss in RIF^11^, however, among these cell types, renal tubular epithelial cells (TECs) stand out as critical mediators of this process. TECs possess the remarkable ability to initiate an adaptive response to injuries, such as ischemia or hypoxia, enabling them to fully recover from mild acute kidney injury (AKI) due to their superior regenerative capacity. In contrast, severe AKI and persistent CKD lead to maladaptive repair processes, ultimately contributing to RIF^12^. The cellular response of TECs to hypoxia, a significant stressor, involves a key molecular pathway mediated by hypoxia-inducible factors (HIFs). HIFs consist of α subunits, primarily HIF-1α and HIF-2α, whose expression is oxygen dependent, along with a constitutively expressed beta subunit known as HIF-1β or ARNT^13^.

RNA sequencing combined with bioinformatics constitutes an effective and systematic approach to comprehensively profile numerous transcriptomic alterations within genomic information^14^. Furthermore, through bioinformatics analysis, Liu et al. established a dependable prognostic model for diabetic kidney disease (DKD) comprising eight ferroptosis-related genes (FRGs)^15^. Hence, comprehensive bioinformatics analyses to identify novel markers of angiogenesis under hypoxia in RIF hold significant value.

**Figure 1:**
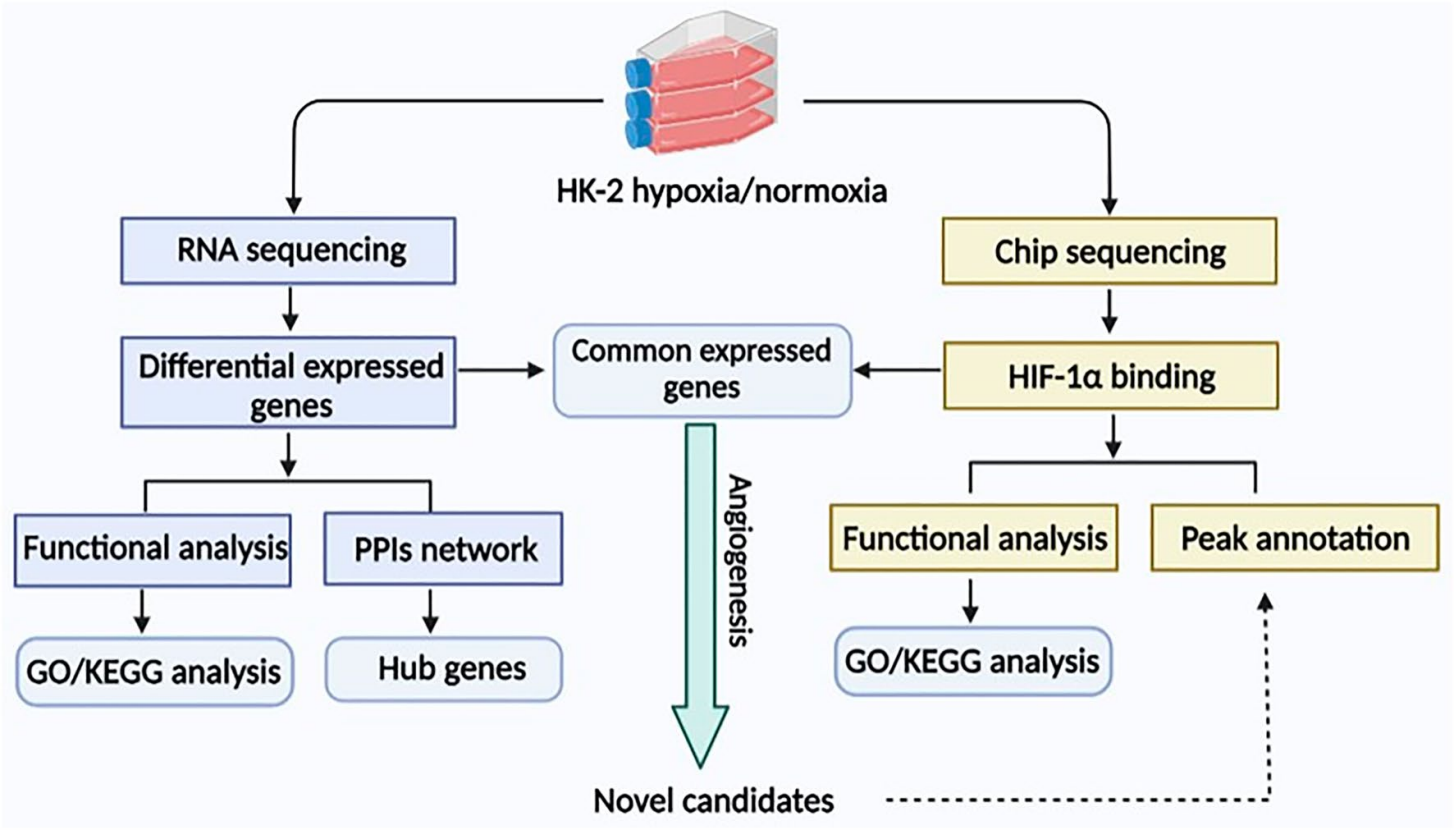
Study workflow. Transcriptomic data were collected through RNA sequencing, and transcriptional alterations in HK-2 cells between hypoxic and normoxic conditions were compared. Functional characterization, including GO analysis and KEGG enrichment, was performed, and hub genes in the PPI network were subsequently identified with Cytoscape software. In parallel, ChIP sequencing was utilized to identify the genes bound by HIF-1α. Importantly, novel candidates were selected from genes that were common to both datasets and functionally annotated as associated with angiogenesis.

In this study, we established hypoxic cell culture models of HK-2 cells (human kidney 2 cells, also known as human renal tubular epithelial cells) and captured myriad novel differentially expressed genes (DEGs) through RNA sequencing. KEGG pathway enrichment analysis revealed that the HIF-1 signaling pathway played a prominent role in this process. Thus, chromatin immunoprecipitation (ChIP) sequencing targeting HIF-1α was implemented to elucidate the underlying DNA‒protein interaction mechanisms. We integrated transcriptome data with ChIP sequencing and identified biological transcripts targeting hypoxia in a HIF-1α-dependent manner. Ultimately, the results were further validated through functional analysis specific to angiogenesis and peak annotations, thereby enabling us to discover relevant molecules. A schematic of the workflow of this research is shown in Fig. 1. Our study may offer valuable insights for further biological mechanistic studies and help discover novel therapeutic targets.

## Materials and methods

### Human specimen collection & ethics approval

31 Specimens of CKD and 14 normal kidney tissues adjacent to the tumor were collected from the Institute of Nephrology, Zhongda Hospital, and the Department of Pathology, Zhongda Hospital, School of Medicine, Southeast University. For this study, we applied the following inclusion/exclusion workflow: (i) 31 cases of histopathologically diagnosed chronic kidney disease (CKD) and 14 patients affected by a pathology proven healthy tissue adjacent to the tumor, (ii) demographic records including age and sex (details were shown in Table S1 of Supplement 1) and pathological diagnosis were available and (iii) inadequate amounts of formalin embedded tissue was excluded from the study.

An informed consent exemption was granted by the Independent Ethics Committee (IEC) for Clinical Research of Zhongda Hospital, Southeast University (2019ZDKYSB162), and all methods in this study were conducted in accordance with relevant guidelines and regulations.

### Immunohistochemistry (IHC)

CD34 staining was performed following the manufacturer's protocol (Abcam). Tissue sections were incubated with primary anti-CD34 antibody (1:200, ab110643, Abcam, USA) at 4 °C overnight, and we employed tissues that did not undergo primary antigen treatment as negative controls. These negative control tissues were processed in parallel with the experimental tissues, following the same procedures, except for the omission of the primary antibody incubation step. Then tissues were incubated with a corresponding secondary antibody for 30 min at room temperature. Digital images were scanned using OlyVIA software (v3.2, Olympus). For quantification analysis, an experienced pathologist selected at least five random fields of the same size in the cortical areas under light microscopy, with verification by another pathologist. Any discrepancies were resolved through discussion and consensus among the observers**.** The microvessel density in this study was defined as the average number of CD34-positive intact capillary lumens observed in every 5 sections of renal cortical sections according to previous research (Magnification, 200×)^16^.

### Sirius red staining

CKD specimens were stained with Sirius red (Senbeijia, Nanjing, China) for 30 min. Fibrosis was defined as the presence of collagen fibers that appeared red under a light microscope^17^, which were evaluated by ImageJ software (National Institutes of Health, Bethesda, Maryland, USA).

### Cell culture and treatments

Human kidney-2 (HK-2) cells, also known as human renal tubular epithelial cells, were purchased from Zhongqiaoxinzhou (Shanghai, China). DMEM/F-12 (1:1) basic (Gibco, USA) with 10% fetal bovine serum (FBS) was used for the HK-2 cell culture.

The HK-2 normoxia group was incubated at 37 ℃ in an incubator containing 5% CO_2_ and 21% O_2_ for 24 h. For hypoxia treatment, HK-2 cells were cultured in an atmosphere of 5% CO_2_, 94% N_2_ and 1% O_2_ for 24 h or 6 h at 37 ℃ as described previously^18,19^.

### RNA extraction and real-time quantitative PCR (RT‒qPCR)

Total RNA was extracted separately from all groups with Total RNA Extraction Reagent followed manufacturer's protocol (Vazyme, Nanjing, China). RNA samples with high purity and integrity (OD_260/280_:1.9–2.0) were selected for downstream experiments. Subsequently, RNA reverse transcription was performed using HiScript III RT SuperMix (Vazyme, Nanjing, China). qPCR was carried out using the Tap Pro Universal SYBR qPCR Master Mix (Vazyme, Nanjing, China). mRNA expression levels were detected on StepOne software v2.3. The final results were normalized with respect to β-actin. The primer sequences were designed by Sangon Biotech (Shanghai, China) and are described in Table S2 of Supplement 1. All experiments were independently repeated at least 3 times.

### RNA sequencing

RNA sequencing was carried out by Nanjing Geneseeq Technology. The RNA purity was assessed using a NanoPhotometer spectrophotometer (IMPLEN, USA), and the RNA concentration was measured using a Qubit 2.0 Fluorometer (Life Technologies, USA). RNA integrity was evaluated using the Bioanalyzer 2100 system (Agilent Technologies, USA). The sequencing libraries were generated and subsequently sequenced using the Illumina HiSeq 4000 platform.

### Identification of DEGs

Data filtering was performed using Trimmomatic software (v0.36). The resulting clean reads were aligned to the reference genome using Spliced Transcripts Alignment to a Reference (STAR, v2.5). The expected number of fragments per kilobase of transcript sequence per million base pairs sequenced (FPKM) in each sample was calculated using RSEM (v1.1.17). Multiple testing corrections such as Bonferroni and Benjamini–Hochberg were used to control the false discovery rate (FDR) to obtain *p*‐adjust in the analysis of DEGs. The transcripts with |log_2_(FC) |> 1 and *p*‐adjust < 0.05 were assigned as DEGs, which were analyzed by DESeq2 software (v1.16.1).

### Functional analysis of DEGs

Gene Ontology (GO) term enrichment was analyzed using the topGO package (v2.28.0), while pathway enrichment was examined using Kyoto Encyclopedia of Genes and Genomes (KEGG) analysis (http://www.genome.jp/kegg). These analyses were performed to explore the potential functions and associated pathways of the DEGs.

### Hub gene identification

To construct the protein‒protein interaction (PPI) networks, we utilized the Search Tool for the Retrieval of Interacting Genes (STRING, v10.5) (http://www.stringdb.org/). The resulting PPI networks were visualized using Cytoscape (v3.7.1) software. Any single differentially expressed protein that could not form an interaction relationship or had only two nodes with one edge was removed from the network.

To identify the significant proteins within the PPI network, we utilized the cytoHubba plugin in Cytoscape to calculate fragile motifs^20^. In this study, we utilized five well-established algorithms, including closeness, degree, EPC, MCC, and MNC. These algorithms are recognized for their effectiveness in identifying central nodes, utilizing various criteria such as network connectivity, centrality, and influence to extract hub genes from the PPI network^21^. The hub genes in this study were defined as common central modules that were consistently prominent in all algorithms.

### ChIP sequencing

HK-2 cells (5 × 10^7^ cells) were cultured for 6 h under either hypoxic or normoxic conditions. Subsequently, the cells were fixed with 1% formaldehyde at room temperature for 10 min, and the fixation reaction was quenched with 2.5 M glycine (1:20) for 5 min. The cell precipitates were then collected and centrifuged at 1200 r/min and 4 °C for 3 min. The cell precipitates were collected and centrifuged at 1200 r/min and 4 °C for 3 min. Subsequently, the collected cell precipitates were sonicated to generate DNA fragments. The chromatin fragments were then immunoprecipitated and incubated at 4 °C overnight using antibodies against HIF-1α (ab243860, Abcam). Magnetic Protein G beads (50 µl) were placed into 1.5 ml EP tubes and mixed with ChIP binding buffer. The incubation was performed until the liquid became transparent. Subsequently, the chromatin fragment–antibody mixture was added to the magnetic beads and incubated for 4 h at 4 °C. The precipitated DNA was then purified using the ChIP DNA Clean Kit.

ChIP sequencing was carried out by KeyGEN Bio TECH (Nanjing, China) using an Illumina NovaSeq 6000. The reads were aligned to the reference genome (hg38) using Bowtie2. Peak calling and annotation were performed using Hypergeometric Optimization of Motif Enrichment (HOMER). Redundant reads were removed from the comparison files, and visualization was performed using IGV software.

## Results

### Peritubular capillary rarefaction and hypoxia in RIF

**Figure 2:**
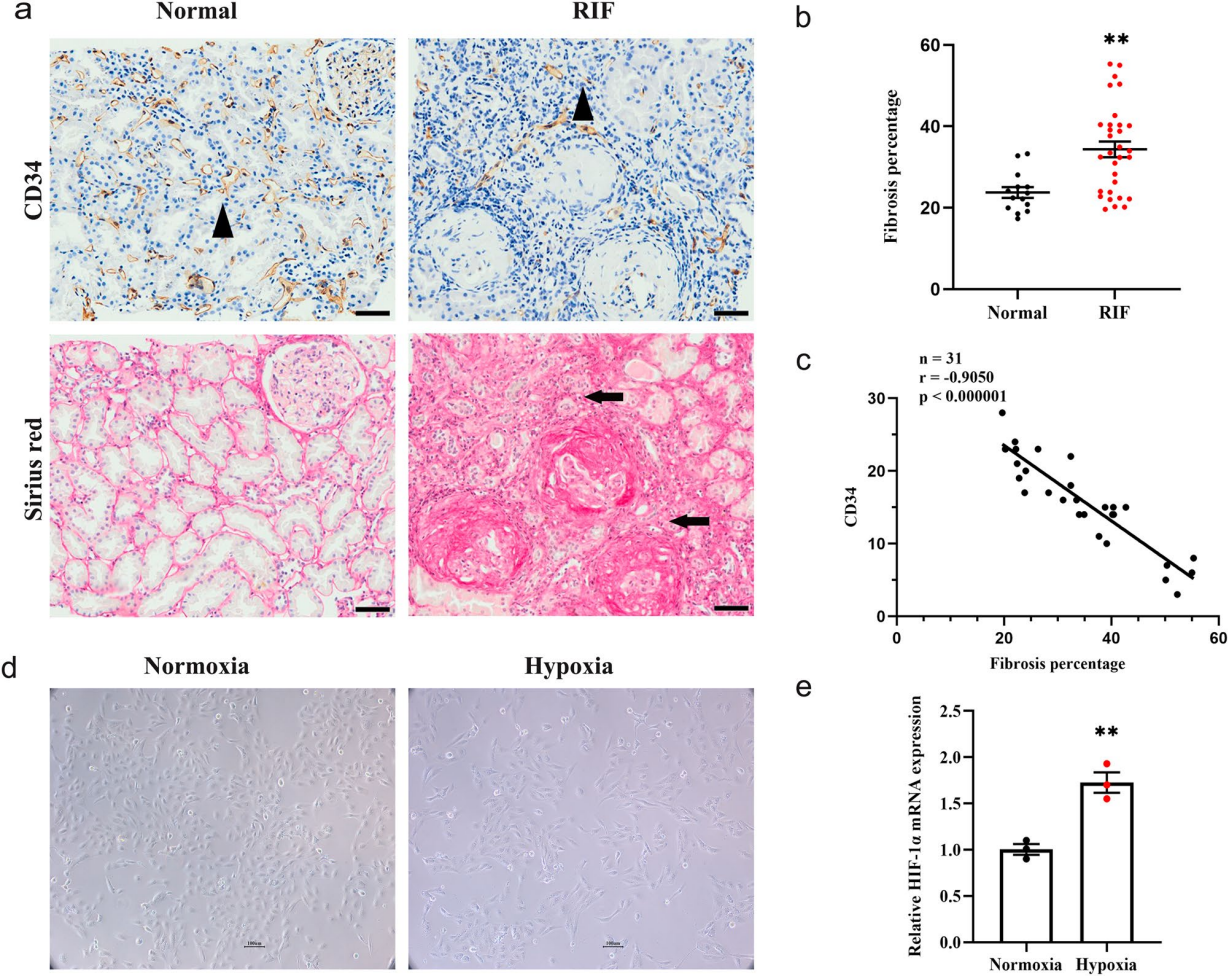
Peritubular rarefaction and hypoxia in RIF. (**a**) CD34 positivity as assessed by IHC staining was definitely decreased in RIF compared with normal tissue. Triangles indicate typical microvessels, while the arrows show tubular interstitial fibrosis. Magnification, 200X. Scale bars, 20 μm. (**b**) Statistical analysis of fibrosis percentage comparation among normal and RIF. (**c**) Correlation between fibrosis degree and microvessel density with CD34 positivity in RIF. (**d**) Cell morphology of established HK-2 hypoxia models at 24 h. Scale bars, 100 μm. (**e**) RT‒qPCR validated the increase in HIF-1α mRNA expression in response to hypoxia.

The results of Sirius red staining, as shown in Fig. 2a, clearly revealed a significantly higher ratio of collagen deposition in the RIF specimens than in the normal group (Fig. 2b). Upon combination with the microvessel density analysis, we observed a significant decrease in CD34 staining positivity in RIF patient specimens compared to normal specimens, indicating a remarkable loss of peritubular capillaries (PTCs) in the RIF kidneys. Additionally, the results of the simple linear regression analysis presented in Fig. 2c demonstrated an inverse relationship between microvessel density and the degree of fibrosis in RIF. To simulate hypoxia conditions and investigate the associated molecular changes in RIF, we established a hypoxic HK-2 cell model. Images depicting HK-2 cells cultured under normoxic and hypoxic conditions (Fig. 2d) provided visual confirmation of the successful induction of hypoxia. This was further supported by the increase in HIF-1α mRNA expression levels following hypoxic treatment, which served as validation for the effectiveness of our model (Fig. 2e).

### Identification of DEGs

**Figure 3:**
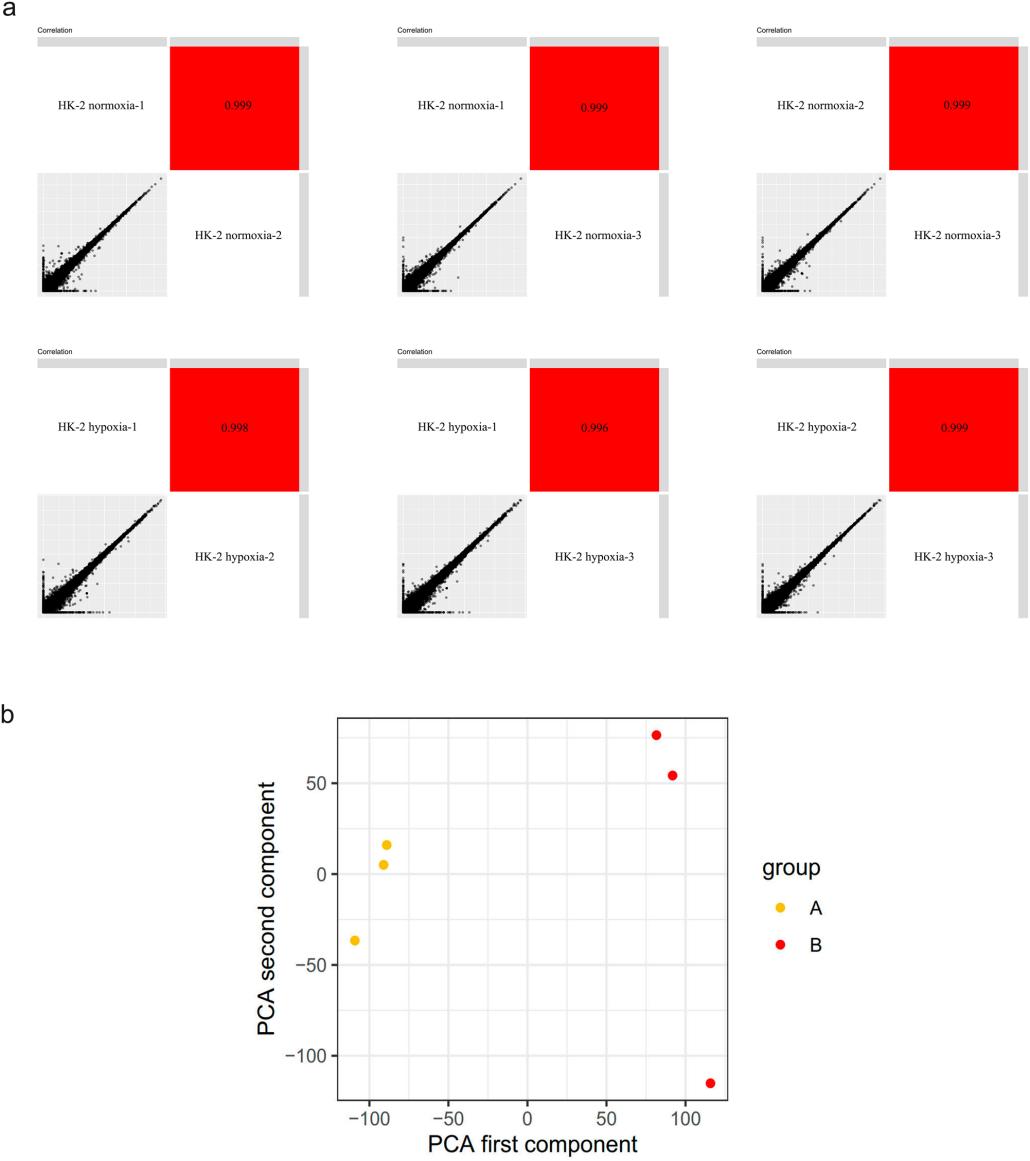
Data quality assessment. (**a**) Scatter plots show predominant correlations between the expression levels of multiple mRNAs among samples. (**b**) PCA showed that the samples clustered into two groups. A: HK-2 normoxia, B: HK-2 hypoxia.

**Figure 4:**
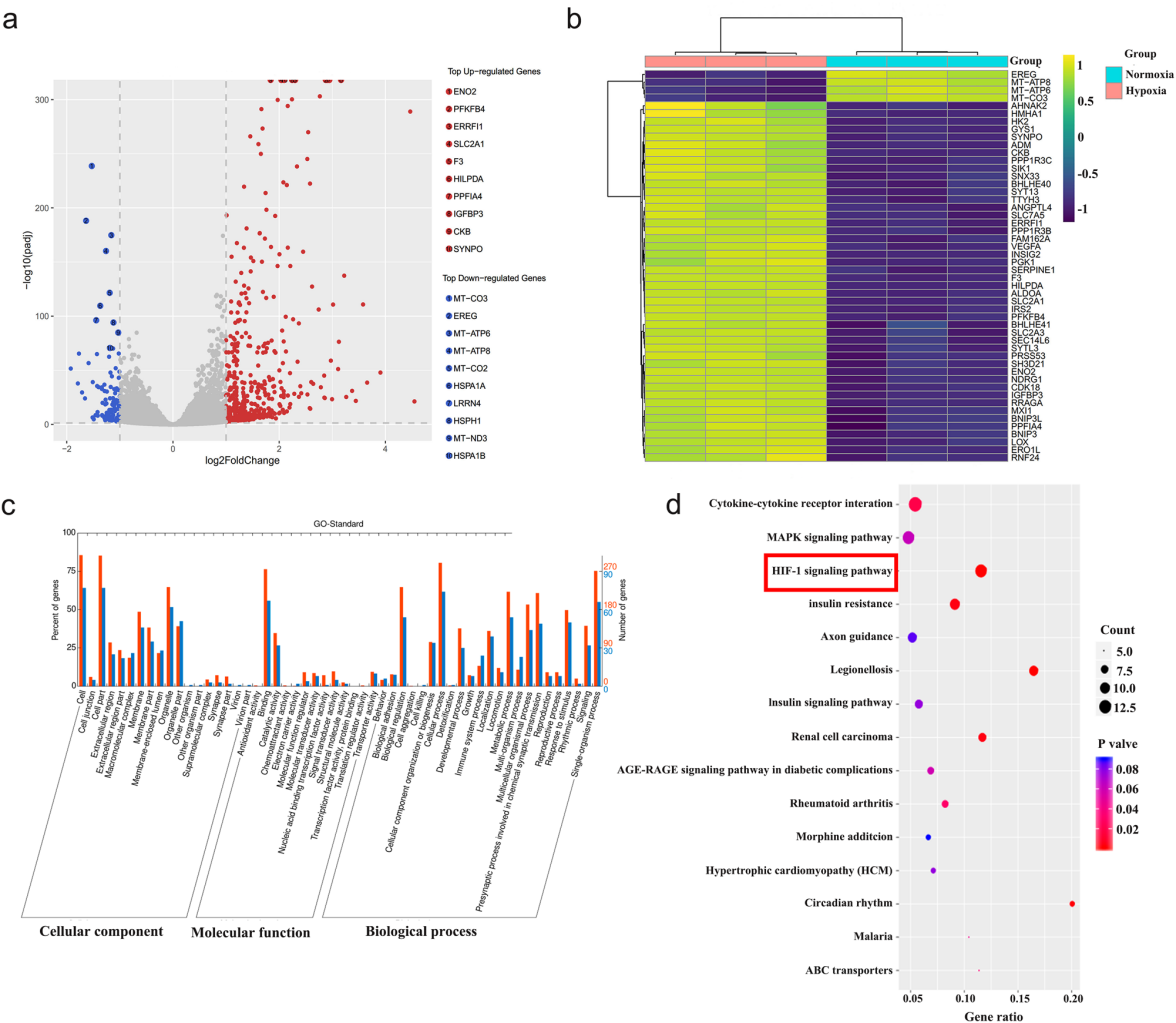
Analysis of DEGs in HK-2 normoxia compared with hypoxia. (**a**) Volcano plots of DEGs between the two groups. The cutoff criteria for significant DEGs were *p*-adjust < 0.05 and |log_2_(FC)|> 1. The significantly upregulated transcripts and downregulated transcripts are shown in red and blue, respectively. (**b**) Heatmaps indicating hierarchical clustering of DEGs between HK-2 normoxia and HK-2 hypoxia. The color from green to blue indicates upregulation to downregulation. (**c**) GO analysis of total DEGs. (**d**) KEGG enrichment analysis of total DEGs, *p*-adjust < 0.05.

As seen in the scatterplots (Fig. 3a) and PCA (Fig. 3b), the samples clustered into two groups, with one outlier. While we acknowledge the presence of heterogeneity in vivo, we believed it essential to include this outlier in our subsequent analysis to represent the variability that can occur in real-world scenarios. A total of 572 DEGs were identified from the HK-2 hypoxia vs HK-2 control profile datasets, indicating numerous biological alterations in RIF. The top 10 significantly upregulated and downregulated genes, ranked by *p*-value, in response to hypoxia when compared to normoxia were presented in Fig. 4a. Furthermore, Fig. 4b illustrated a hierarchical clustering analysis of the DEGs. These comparisons at the cellular level revealed substantial alterations in gene expression under hypoxia, supporting the hypothesis that multiple genes contributed to the differential angiogenesis state observed in vivo among RIF.

### GO function and KEGG pathway enrichment of DEGs

To gain deeper insights into the specific biological functions and signaling pathways associated with the identified DEGs, we conducted Gene Ontology (GO) analyses, as illustrated in Fig. 4c. The GO analysis revealed significant enrichment across various categories. In terms of cellular components (CC), the analysis highlighted 'cell,' 'cell junction,' and 'organelle' as the top three enriched categories. These findings underscored the importance of these cellular structures in the context of the studied DEGs. Regarding molecular function (MF) annotations, the GO analysis pointed to 'binding' and 'catalytic activity' as the most pivotal functions among the DEGs. These molecular functions likely played key roles in the regulation of cellular processes. Our particular focus in the biological processes (BP) category centered on angiogenesis, a critical process in the context of RIF. Within this framework, the analysis revealed that three genes (GO:0045766, *p*-value: 0.009) exhibited positive regulation of angiogenesis in hypoxic HK-2 cells. Additionally, most of the genes in the same category (GO:0001525, *p*-value: 0.002; GO:0045766, *p*-value: 0.002) were found to be downregulated, suggesting a complex and context-dependent regulation of angiogenic processes in response to hypoxia.

The KEGG analysis (Fig. 4d) unveiled substantial enrichment in pathways, with their significance levels indicated by *p*-values. Among these pathways, the HIF-1 signaling pathway exhibited the highest degree of enrichment (*p*-value < 0.001), followed by insulin resistance (*p*-value: 0.004) and cytokine-cytokine receptor interaction (*p*-value: 0.020).

### PPI network analysis and hub gene identification

**Figure 5:**
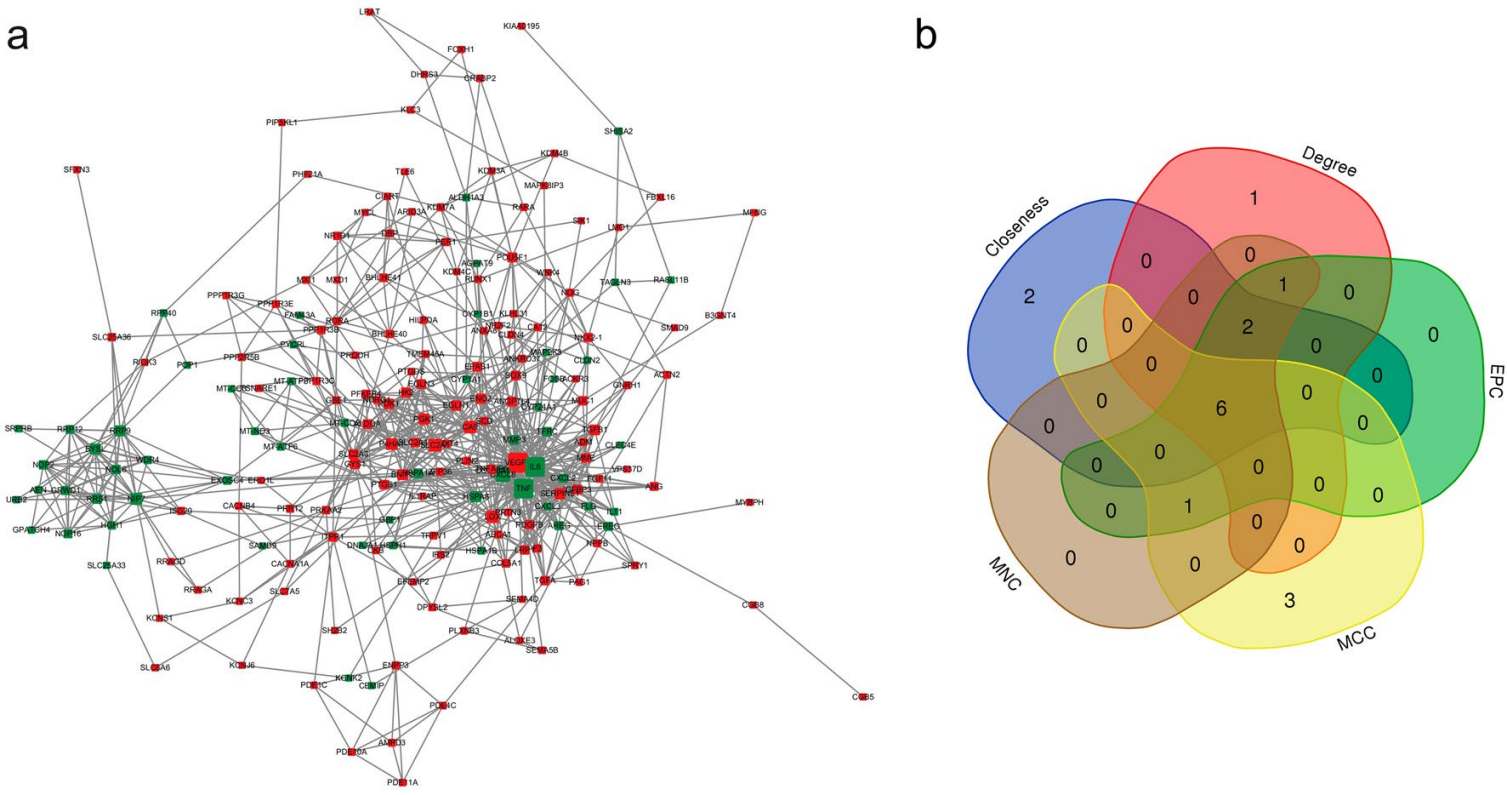
PPI network and hub gene analysis reveal the interactions among the DEGs. (**a**) PPI analysis of total DEGs. Proteins shown in red color were upregulated, and proteins shown in green were downregulated. The size of the dot is proportional to the number of interacting proteins. (**b**) Venn diagram illustrating the hub genes involved in these networks, which were predicted and explored with five topology algorithms.

We constructed and visualized the PPI network of the DEGs to identify their potential functions, which were either environmentally determined or predicted. All 572 DEGs between normoxic and hypoxic HK-2 cells were utilized, resulting in the selection of 347 genes for the construction of the PPI network (Any single differentially expressed protein that could not form an interaction relationship or had only two nodes with one edge was removed from the network). As depicted in Fig. 5a, it was noteworthy that the majority of these interacting proteins (275 out of 347 genes) exhibited enhanced expression in response to hypoxia.

To unravel the crucial pathogenic mechanisms of RIF in humans, it is crucial to explore key proteins and their associated subnetworks within this complex PPI network. To achieve this, we employed five algorithms from the cytoHubba plugin in Cytoscape to identify the top 10 genes individually. Through the intersection of Venn diagrams, we ultimately selected six common genes as hub genes, namely, vascular endothelial growth factor A (VEGFA), C-X-C motif chemokine ligand 8 (CXCL8), Lysyl oxidase (LOX), carbonic anhydrase 9 (CA9), interleukin 6 (IL6), and tumor necrosis factor (TNF) (Fig. 5b; details in Table 1).

**Table 1:** Analysis of potential potent genes in response to hypoxia.

Gene	Description	Log twofold change	*p*-adjust	Regulated
VEGFA	Vascular endothelial growth factor A	1.666	5.682e − 292	Upregulated
CXCL8	C-X-C motif chemokine ligand 8	− 1.252	4.599e − 20	Downregulated
LOX	Lysyl oxidase	1.972	1.849e − 300	Upregulated
CA9	Carbonic anhydrase 9	3.656	2.407e − 39	Upregulated
IL6	Interleukin 6	− 1.712	2.840e − 30	Downregulated
TNF	Tumor necrosis factor	− 1.514	3.432e − 07	Downregulated

### ChIP sequencing

**Figure 6:**
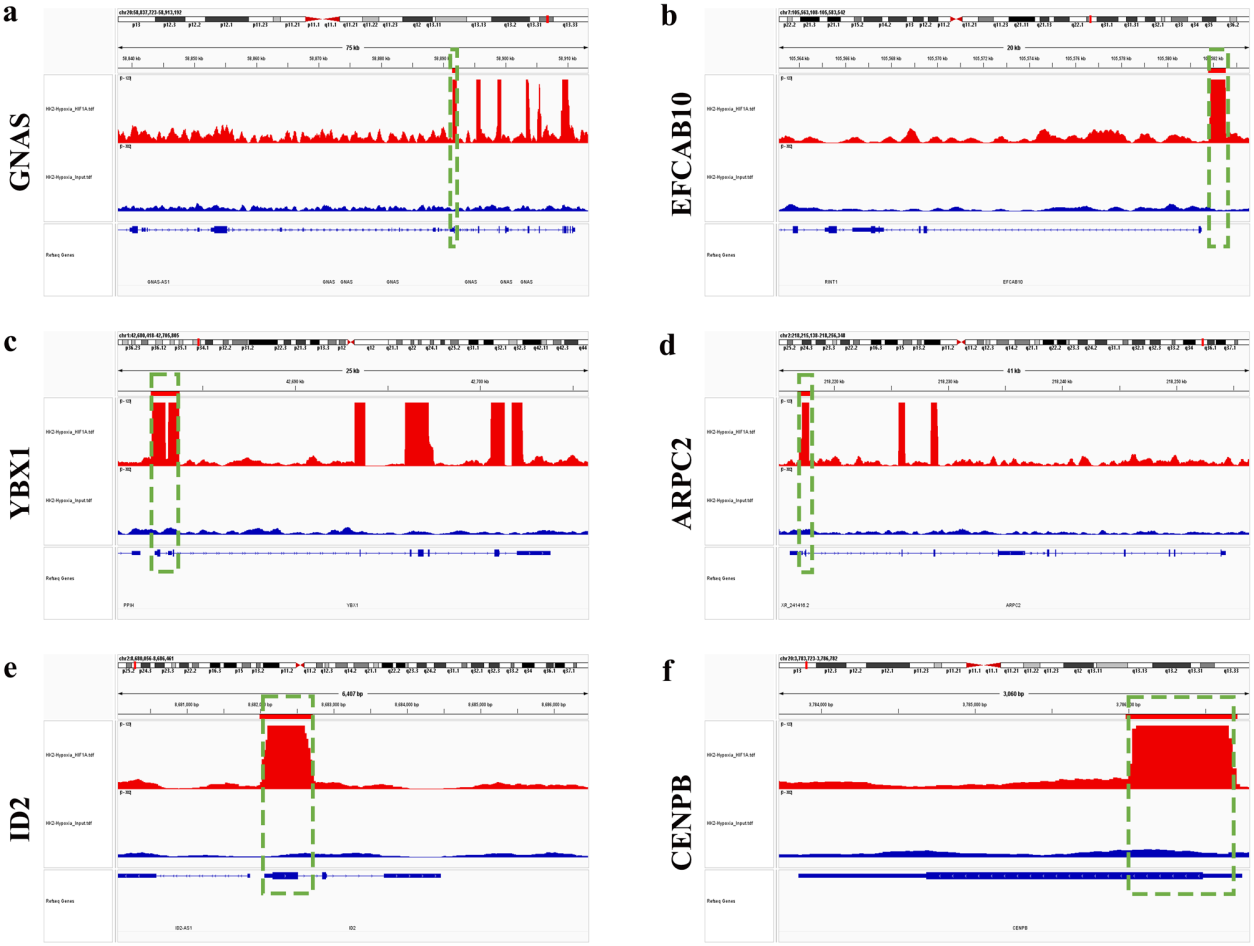
Illustrations showcasing the binding sites at the promoters of the 10 most interacting genes, which include (**a**) GNAS, (**b**) EFCAB10, (**c**) YBX1, (**d**) ARPC2, (**e**) ID2, (**f**) CENPB. These genes were ranked based on their peak scores.

**Figure 7:**
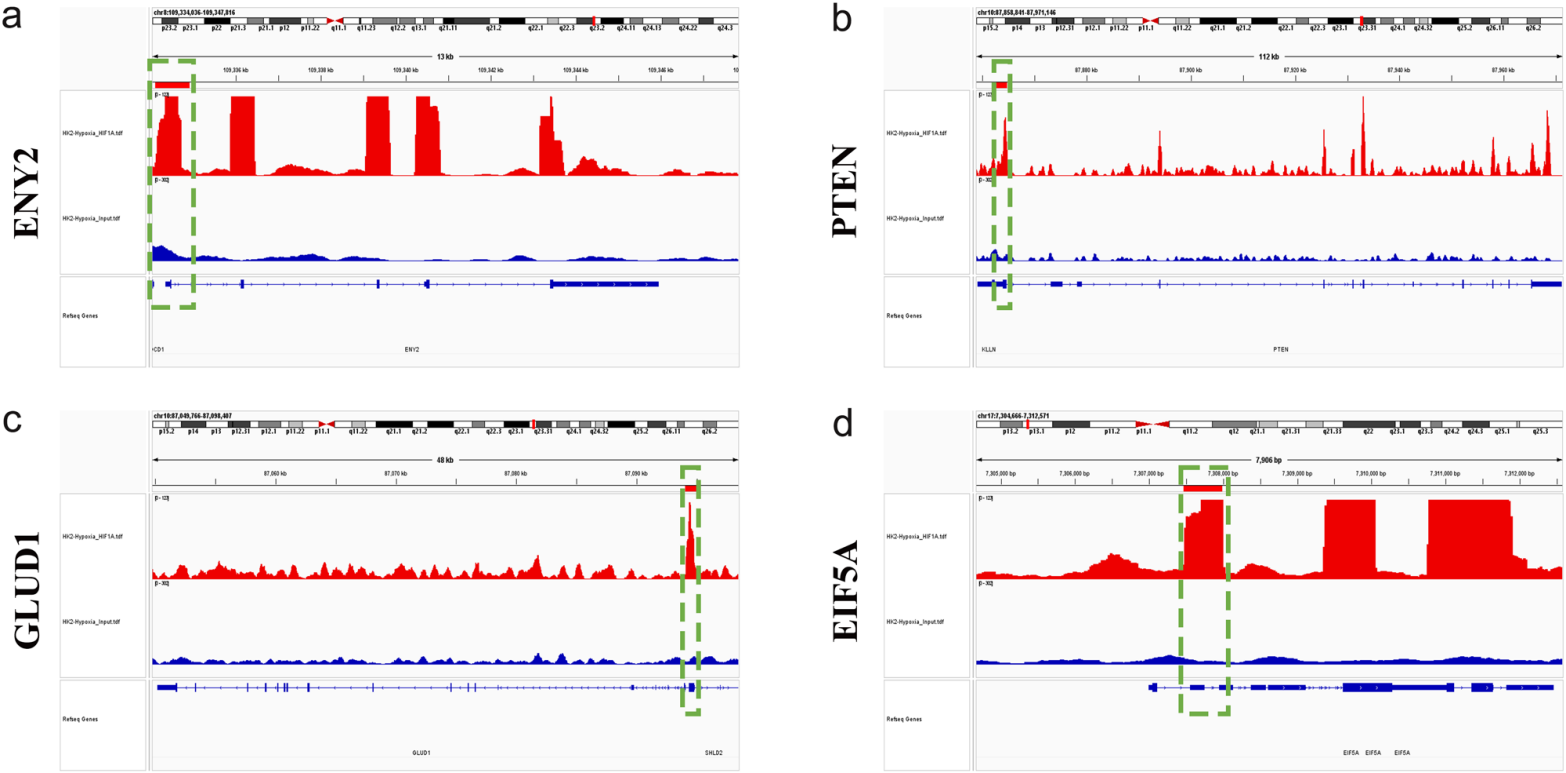
Illustrations showcasing the binding sites at the promoters of the 10 most interacting genes, which include (**a**) ENY2, (**b**) PTEN, (**c**) GLUD1, and (**d**) EIF5A. These genes were ranked based on their peak scores.

We observed an abundance of HIFs in response to hypoxia, with HIF-1α actively expressed during the early phase of hypoxia. HIF-1α acts as a hypoxic transcriptional activator, binding to the hypoxia-responsive element (HRE) to regulate gene expression^22^. Moreover, KEGG analysis of DEGs in RNA sequencing revealed enrichment of the HIF-1α signaling pathway. Therefore, our next investigation focused on identifying genes that could be directly activated by HIF-1α, aiming to discover more prominent biomarkers. To achieve this, we utilized ChIP sequencing to analyze genes that interact with HIF-1α, examining DNA‒protein interactions under hypoxic conditions. A total of 2915 genes were identified (Fig. 9a) by alignment to the reference genome (hg38). Based on peak scores (1/*p*-value), we identified the ten most significant genes, namely, GNAS complex locus (GNAS), EF-hand calcium binding domain 10 (EFCAB10), Y-box binding protein 1 (YBX1), actin-related protein 2/3 complex subunit 2 (ARPC2), inhibitor of DNA binding 2 (ID2), centromere protein B (CENPB), ENY2 transcription and export complex 2 subunit (ENY2), phosphatase and tensin homolog (PTEN), glutamate dehydrogenase 1 (GLUD1), and eukaryotic translation initiation factor 5A (EIF5A). These genes are known to be regulated by HIF-1α through their promoter regions (Figs. 6a–f and 7a–d). We also carefully examined the peak distributions of all genes bound by HIF-1α, as listed in Table 2. We then focused specifically on noncoding RNAs (ncRNAs) (Table 3). The 20 ncRNAs with the most significant HIF-1α binding were presented in Table S3. These findings offered valuable insights into potential future research directions involving ncRNAs. In addition, to verify the reliability of the ChIP sequencing, we also performed ChIP under normoxic conditions, which showed that there were only a few binding sites (Tables S4 and S5 of Supplement 1), consistent with the rapid degradation of HIF-1α under normoxic conditions.

**Table 2:** Distributions of binding sites with HIF-1α under hypoxia.

Annotation	Number of peaks	Total size (bp)	Log2 ratio (obs/exp)	LogP enrichment (+ values depleted)
3UTR	179	26,833,139	2.831	− 204.921
TTS	79	32,404,629	1.379	− 29.914
Promoter	69	35,946,139	1.034	− 16.75
Exon	510	37,120,946	3.873	− 939.718
Intron	1182	1,257,910,936	0.003	− 0.757
5UTR	17	2,601,483	2.801	− 20.676
Intergenic	825	1,684,358,172	− 0.937	406.224

**Table 3:** Annotations of noncoding RNA binding with HIF-1α under hypoxia.

Annotation	Number of peaks	Total size (bp)	Log2 Ratio (obs/exp)	LogP enrichment (+ values depleted)
scRNA	0	97	0	0
snoRNA	0	357	0	0
rRNA	0	25,562	− 0.034	0.024
miRNA	0	97,618	− 0.126	0.092
ncRNA	18	7,044,070	1.446	− 8.627

**Figure 8:**
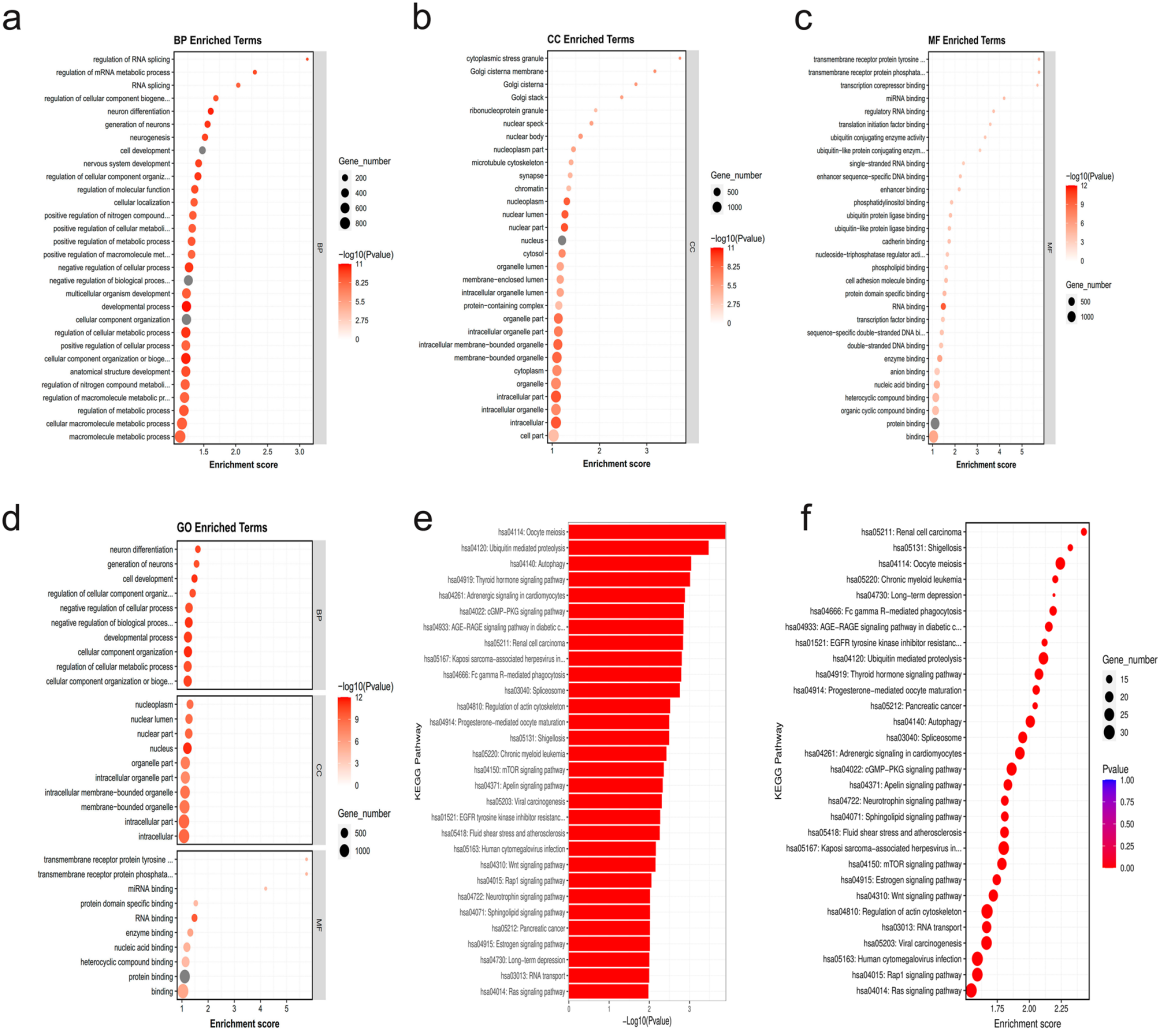
GO and KEGG enrichment of ChIP sequencing findings. (**a**) Enriched biological process terms. (**b**) Enriched cellular component terms. (**c**) Enriched molecular function terms. (**d**) Top 10 GO processes among all subtypes. (**e**) Bar plots of enriched KEGG pathways. (**f**) Dot plots of enriched KEGG pathways.

The results were subsequently subjected to GO and KEGG enrichment analyses. The top 30 enriched BP, CC, and MF terms were shown in Fig. 8a–c, respectively. Additionally, the top 10 terms from all subtypes were displayed in Fig. 8d, ranked by their *p*-values. The GO enrichment analyses of BP terms revealed the top 3 enrichment (*p*-value < 0.0001) in the following categories: macromolecule metabolic process, cellular macromolecule metabolic process, and regulation of metabolic process. As evident from the CC annotations, the top 3 components *(p*-value < 0.0001) were cell part, intracellular, and intracellular organelle. The top 3 MF (*p*-value < 0.0001) annotations included binding, protein binding, and organic cyclic compound binding. Moreover, our KEGG pathway enrichment findings (Fig. 8e, f), suggested that ubiquitin mediated proteolysis (*p*-value < 0.0001), autophagy (*p*-value < 0.0001) and Ras signaling (*p*-value: 0.010) pathways were likely to be significantly involved in HIF-1α-induced hypoxia.

### Genes modulated by HIF-1α

**Figure 9:**
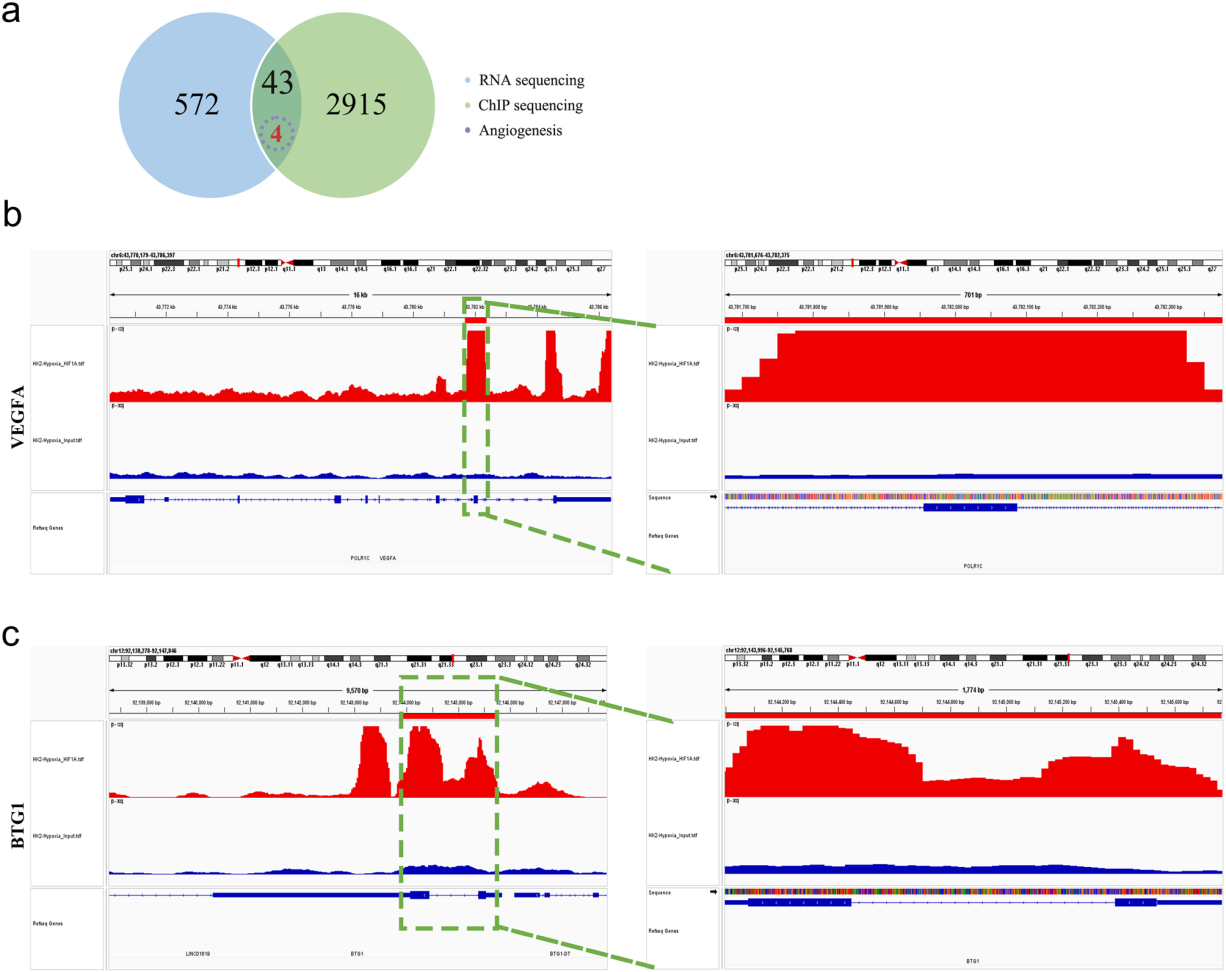
ChIP sequencing for novel candidates. (**a**) Venn diagram illustrating the overlap between the DEG dataset from RNA sequencing and the HIF-1α-interacting genes identified through ChIP sequencing. (**b)** and (**c**) Peak annotations and plausible binding sites in VEGFA and BTG1.

Following the comparison of RNA sequencing and ChIP sequencing data, we identified 43 genes that demonstrated a shared presence in both datasets. These genes exhibited differential expression in response to hypoxia and were targeted by HIF-1α. Subsequently, we prioritized potential genes from this group based on functional analysis, giving preference to those associated with angiogenesis. This process led to the identification of four candidates linked to angiogenesis within the context of renal fibrosis (Fig. 9a). However, to establish a robust foundation for subsequent experiments, we further assessed the biological significance of their binding sites. As a result, only two genes, VEGFA and BTG anti-proliferation factor 1(BTG1), were ultimately designated as novel targets (Fig. 9b, c). (For more detailed information about these genes and their respective binding sites, refer to Table 4.)

**Table 4:** Annotated analysis of candidates.

Gene	Gene description	Chr	Start	End	Strand	Peak score	Annotation
VEGFA	Vascular endothelial growth factor A	Chr6	43,781,625	43,782,536	+	246.54	exon (NM_001171626, exon 6 of 7)
BTG1	BTG anti-proliferation factor 1	Chr12	92,143,996	92,145,768	+	95.16	intron (NM_001731, intron 1 of 1)

## Discussion

The absence of peritubular capillaries is strongly associated with RIF and is a widespread phenomenon observed in CKD patients^23^. In the literature, it has been well-documented that vascular endothelial cells are modulated by autocrine signals, including proangiogenic factors and anti-angiogenic factors^5,23^. Recent studies have also shed light on the separation of pericytes from capillaries as a critical step in capillary thinning^4,23,24^. However, it's worth noting that pro-angiogenesis therapies targeting these factors have shown mixed results and can sometimes lead to adverse effects. For instance, in diabetic nephropathy, VEGF-driven angiogenesis could result in formation of abnormal vessels with loose connections, ultimately triggering tubular interstitial inflammation and reducing capillary flow and inducing hypoxia^6,25,26^. Consequently, despite extensive research, there are currently no effective therapies available to halt CKD progression, underscoring the pressing need for further exploration of novel targets alongside established mechanisms.

The magnitude of hypoxia in CKD, which hampers oxygen delivery and activates fibroblasts and inflammation, has a significant impact on the integrity and quantity of interstitial capillaries in renal fibrosis, further accelerating the progression to ESRD^27^. In CKD, HIFs especially HIF-1α accumulates in the ischemic tubulointerstitium^28^, therefore, the primary objective of this study was to pinpoint novel therapeutic modulators, which were associated with angiogenesis and activated by HIF-1α in the context of tubular hypoxia in RIF. To achieve this, we employed a combined RNA sequencing and ChIP sequencing approach as a preliminary step to identify potential candidate genes that respond to hypoxia and are significantly regulated by HIF-1α. By using this integrated approach, we were able to narrow down the list of candidate genes and prioritize those that were consistently identified by both techniques. Subsequently, through GO analysis, we identified significant targets for angiogenesis among the overlapping genes, as angiogenesis is relevant to the context of RIF.

In this study, RNA sequencing unveiled significant differences in gene expression profiles between hypoxic HK-2 cells and their normoxic counterparts. A total of 572 differentially expressed genes (DEGs) were identified, with the majority (469 genes) demonstrating upregulation, while only 103 were downregulated. Moreover, in the PPI network, a notable observation was that the majority of these interacting proteins (275 out of 347 genes) exhibited enhanced expression in response to hypoxia. This observation aligns with a previous study that reported 3131 differentially expressed transcripts in extracellular vesicles (EVs) from renal tubular epithelial cells, the majority of which exhibited heightened expression under hypoxic conditions^29^. These findings underscore the sensitivity of most DEGs to hypoxia and their activation during this process. Notably, the most highly upregulated gene shown in Fig. 4a in response to hypoxia was Enolase 2 (ENO2). This gene encodes one of the three enolase isoenzymes present in mammals and is associated with glycolysis. While there is limited research on its role in CKD, it has been linked to reliable functions in retinal angiogenesis in murine neonates^30^.

The KEGG enrichment analysis of DEGs in this study highlighted the significance of insulin resistance and cytokine-cytokine receptor interaction and the HIF-1 signaling pathway. Cytokines play crucial roles in various biological functions, including immunity, tissue regeneration, and metabolism, exhibiting a wide variety of effects. Cytokine‒cytokine receptor interactions allow cells to communicate with and influence neighboring cells through cytokine networks, which involve cytokine production and secretion and the recruitment of immune cells such as macrophages, mast cells, and other mesenchymal progenitor cells (MPCs)^31,32^. Consistent with these results above, by employing the novel hub score measures from the cytoHubba plugin, we identified several central modules that were prominent in all candidates from complex PPIs network, comprising the cytokines CXCL8, IL6, and TNF. While specific studies on these genes in the context of RIF are limited, we have identified novel findings related to these genes in other diseases, which might contribute to the observed effects in the context of angiogenesis and RIF. CXCL8 is a potent chemokine that attracts human neutrophils and promotes angiogenesis by stimulating endothelial cell proliferation and migration to facilitate the repair of damaged tissue^33^. In an animal experiment investigating Cr (VI)-induced carcinogenesis and angiogenesis, miR-199a overexpression was found to inhibit angiogenesis by suppressing IL8, HIF-1α, and NF-κB p65 expression in vivo^3^. IL-6, a cytokine released by monocytes and macrophages, signals directly to microglia, promoting the generation of repair-associated microglia (RAM). This process facilitates successful cerebrovascular repair and contributes to functional recovery^34^. Therefore, CXCL8 and IL-6, identified through hub gene analysis, may represent novel targets that warrant further exploration and investigation.

Moreover, our analysis revealed significant enrichment of the HIF-1 signaling pathway in tubular epithelial cells under hypoxic conditions, a finding shown in our KEGG analysis results (Fig. 4d). This enrichment signifies the activation of downstream signaling pathways that likely play a critical role in modulating the survival of vascular endothelial cells. The role of HIF-1α in angiogenesis within RIF is dependent on oxygen concentration and time, as a transition from HIF-1 to HIF-2 occurs with prolonged hypoxia^31,35^. Existing research suggests that HIF-1 primarily responds to acute hypoxia and plays a significant role in inducing angiogenesis. Different from HIF-1, HIF-2 is more commonly associated with chronic hypoxia and may have varying effects on angiogenesis^28^. This dynamic interplay and transition between HIF-1 and HIF-2 are crucial factors in the progression of RIF. In this study, our primary focus was on HIF-1α, as it is traditionally associated with responses to acute hypoxia and early-stage RIF. However, it's worth noting that HIF-2α's potential role in inversely regulating fibronectin expression in TECs presents an intriguing avenue for further investigation^36,37^. Exploring the modulation of HIF-2α could offer a promising direction for CKD treatment, particularly by mitigating renal damage induced by persistent hypoxia, a hallmark of advanced CKD stages.

Building upon our exploration of RNA sequencing data, we proceeded to investigate proteins activated by HIF-1 using ChIP sequencing. KEGG enrichment analysis of ChIP sequencing revealed several enriched pathways, such as the Ubiquitin mediated proteolysis, Autophagy and Ras signaling. Among these pathways, the Ras signaling pathway emerged as particularly noteworthy. As depicted in Figs. 6a and 7b, the most significantly interacting genes, including GNAS and PTEN, are involved in regulating the Ras pathway and its downstream signaling cascades. The G protein α subunit encoded by GNAS plays a vital role in the Ras signaling pathway. Upon activation of G protein-coupled receptors (GPCRs) on the cell surface by external signals, the G protein α subunit binds to the receptor and catalyzes the exchange of GTP and GDP through its GTPase activity, leading to the activation of the G protein α subunit^38^. Once activated, G protein α subunits promote the binding of GTP to Ras proteins (RAS-GTP) and the dissociation of GDP from Ras proteins (RAS-GDP), resulting in the activation of Ras proteins. Activated RAS-GTP then interacts with downstream signaling molecules to further activate the Ras signaling pathway and initiate a cascade of cell biological responses, including cell proliferation, differentiation and pro-survival pathways^39^. However, specific research on whether GNAS responds to HIF-1-induced hypoxia and regulates angiogenesis in RIF through Ras signals has not yet been conducted. PTEN, as a protein tyrosine phosphatase, plays a key role in dephosphorylating phosphatidylinositol triphosphate (PIP3) on the cell membrane, leading to its conversion into phosphatidylinositol diphosphate (PIP2)^40^. Through this activity, PTEN effectively inhibits the activation of the Ras signaling pathway by suppressing the production of PIP3. Previous studies have reported that hypoxia induces an increase in HIF-1α, which leads to the downregulation of PTEN and upregulation of VEGFA in hepatocellular carcinoma^41^. Based on these findings, we hypothesized that the activation of HIF-1α in RIF may regulate PTEN expression, leading to the negative regulation of Ras signaling and subsequent modulation of VEGFA.

To identify critical therapeutic modulators, we integrated RNA sequencing and ChIP sequencing data. We observed a moderate overlap of genes identified by the two techniques, which significantly narrowed the pool of candidate genes and provided a strong basis for efficiently identifying key genes. Subsequently, we conducted angiogenesis analysis, further narrowing our candidate pool to four genes: VEGFA, BTG1, EGL-9 family hypoxia inducible factor 1(EGLN1), and coagulation factor III, tissue factor (F3). Although this reduced the number of candidates, it posed challenges in terms of extracting hub genes through PPI network. Nonetheless, the integration of these datasets allowed us to gain valuable insights into potential molecular alterations related to angiogenesis in RIF under hypoxic conditions, and further investigations will help us explore the roles of these genes in regulating angiogenesis in the context of renal interstitial fibrosis. Ultimately, our analysis led us to identify two promising candidates (VEGFA and BTG1) through peak annotations, highlighting their significant roles in modulating angiogenesis in response to HIF-1α at reliable genomic regions.

During normal angiogenesis, a multitude of growth factor and cytokine signaling pathways orchestrate the regulation of endothelial cell proliferation, migration, and lumen formation. Notable among these pathways are VEGFA and angiopoietin^5,7^. VEGFA, in particular, emerged as one of the most potent regulators involved in maintaining PTC, with VEGFA expression and secretion observed in tubule epithelial cells^7^. In this study, sequencing data revealed that hypoxia-induced upregulation of HIF-1α in TECs resulted in the activation of VEGFA at exon 6 (NM_001171626, exon 6 of 7). Then, VEGFA binds to and activates VEGF receptor 2 (VEGFR2), thereby promoting endothelial cell survival through transmission of signaling from the interstitial region to the peritubular capillary lumen^7^. Hence, our study's contribution lies in unraveling the specific role of VEGFA in the context of RIF and its interactions with HIF-1α. It is also worth noting the strong response of well-known genes such as VEGFA to hypoxia in fibrosis^42,43^, which adds credibility to our sequencing results and suggests potential avenues for further exploration of the expression and function of our candidates in vivo in future studies.

Our data also revealed a potential association of BTG1 with angiogenesis in RIF, as BTG1 showed a HIF-1α-dependent response to hypoxia, particularly at intron 1 (NM_001731, intron 1 of 1). Initially, BTG1 was found to be associated with chromosomal translocations in B-cell leukemia, but subsequent studies have demonstrated its expression in various cell types, where it plays a regulatory role in cell proliferation, apoptosis, differentiation and metabolism^44,45^. Notably, research on breast cancer has shown that BTG1 expression is significantly associated with hypoxia^46^, aligning with our sequencing results. Additionally, a recent study reported that BTG1 may function as a negative regulator of angiogenesis in brain glioma^47^. These findings call for further in vivo investigations to better understand the role of BTG1 in angiogenesis and its regulation by HIF-1α under hypoxic conditions in the context of RIF. As we move forward, we will expand our exploration of BTG1 to shed light on its potential as a key modulator of angiogenesis and its relevance in the pathogenesis of RIF. To further elucidate the exact mechanisms through which BTG1 regulate angiogenesis in RIF, we will conduct mechanistic studies. This may involve exploring their interactions with other proteins, signaling pathways, or epigenetic modifications that influence their activity. Building on the identification of BTG1 as potential therapeutic targets, we plan to explore novel therapeutic strategies that leverage these molecules. This could involve the development of targeted therapies or drug candidates that modulate their activity to promote angiogenesis in RIF.

Furthermore, our study identified HIF-1α binding not only at promoters but also in intron and exon regions. To address this gap in understanding, we intend to explore novel epigenomic modifications that may contribute to the observed differences in gene expression profiles. Second, as demonstrated in this study (Table 3), various ncRNAs have been implicated in the context of hypoxia-induced fibrosis. We emphasize the necessity for additional exploration of ncRNA-mediated regulatory mechanisms. These molecules have displayed significant promise as potential therapeutic targets in fibrotic disorders, and therefore, they merit comprehensive investigation to fully elucidate their roles and therapeutic potential^48,49^.

In conclusion, our comprehensive analysis, which combined transcriptomics and ChIP sequencing in vitro, has provided valuable insights into the potential molecular alterations involved in PTC loss, particularly under hypoxic conditions, in RIF. The discovery of BTG1 as a potential regulator of angiogenesis in RIF introduces a novel aspect to our research. Modulating these genes or their downstream pathways may present innovative strategies to counteract renal fibrosis, particularly by promoting angiogenesis and enhancing the kidney's microvascular environment. These findings imply the potential for combination therapies that target multiple pathways implicated in CKD, encompassing those related to fibrosis and angiogenesis. Combining treatments aimed at modulating VEGFA, BTG1, and other pertinent targets might offer a more effective approach to decelerating or reversing disease progression. Moreover, comprehending the roles of VEGFA and BTG1 in renal fibrosis may open the door to personalized treatment approaches. Patients with CKD could potentially undergo genetic profiling to determine whether these genes significantly influence their condition, enabling the design of more tailored therapies. We must acknowledge the limitations of our study. Our reliance on bioinformatics analyses presents an inherent risk of false-positive results. To address this, we employed a stringent approach, combining two comprehensive sequencing methods and applying five well-established algorithms for hub gene identification within the protein–protein interaction network. These measures are known for their effectiveness in reducing the likelihood of false positives. Furthermore, we have incorporated multiple testing corrections into our analysis to account for the challenge of multiple hypothesis testing, thereby enhancing the statistical robustness of our results. However, to ensure the credibility and robustness of our findings, it remains imperative to conduct further analyses and experimental validation, possibly using animal models or clinical samples. This will help bridge the gap between cell culture and the complex in vivo environment, providing more stronger evidence and validation for the conclusions drawn in this study.

## Conclusions

This study employed two robust bioinformatics approaches to shed light on the intricate biological changes linked to hypoxia and peritubular capillary rarefaction in renal fibrosis. Significantly, we uncovered two promising molecular candidates, VEGFA and BTG1. Notably, BTG1 represents an exciting new dimension in our investigation. With its involvement in various cellular processes such as cell proliferation, apoptosis, differentiation, and metabolism, the revelation of its role in angiogenesis regulation within the context of RIF presents a novel discovery. The identification of BTG1 as a potential angiogenesis regulator in RIF opens up new avenues for research and hints at its therapeutic potential. Targeting BTG1 holds promise for future interventions aimed at addressing angiogenesis deficiency in RIF, with the ultimate goal of improving outcomes for individuals with chronic kidney disease.

### Supplementary Information

Supplementary Tables.

## Data Availability

The datasets generated during the current study are available in the Gene Expression Omnibus (GEO) repositories GSE225253 and GSE234719.
