# Supplementary material for: Identifying key genes related to the peritubular capillary rarefaction in renal interstitial fibrosis by bioinformatics
Source: Sci Rep. 2023 Nov 10;13:19611. doi: 10.1038/s41598-023-46934-y (PMC10638415; doi:10.1038/s41598-023-46934-y)
Supplement: Supplementary file 1 — Supplementary Tables. [file 41598_2023_46934_MOESM1_ESM.docx]

| **Table S1. Demographic data of patients with CKD** | | |
| --- | --- | --- |
| **Characteristics** | **Types** | **Number** |
| Sex | Women | 9 |
|  | Men | 22 |
| Age categories | ≤30 | 3 |
|  | 30＜~≤50 | 9 |
|  | ＞50 | 19 |

| **Table S2. Primers Sequence for RT-qPCR** | | |
| --- | --- | --- |
| **Name** | **Primer** | **Sequence (5'-3')** |
| β-actin | Forward | GGCCAACCGCGAGAAGATGAC |
|  | Reverse | GGATAGCACAGCCTGGATAGCAAC |
| HIF-1α | Forward | AGTTCCGCAAGCCCTGAAAGC |
|  | Reverse | GCAGTGGTAGTGGTGGCATTAGC |

| **Table S3. Annotations of top 20 non-coding RNA according to peak score** | | | | | | | | |
| --- | --- | --- | --- | --- | --- | --- | --- | --- |
| Entrez ID | Gene Name | Gene Type | Chr | Start | End | Strand | Peak Score | Distance to TSS |
| 100616387 | MIR3064 | ncRNA | chr17 | 64499574 | 64502373 | + | 3100.00 | -134 |
| 100302254 | MIR1282 | ncRNA | chr15 | 43793465 | 43794191 | + | 1530.38 | -69 |
| 106635684 | SNORD141B | snoRNA | chr9 | 133019420 | 133020527 | + | 1367.33 | -457 |
| 692223 | SNORD97 | snoRNA | chr11 | 10801554 | 10802892 | + | 909.73 | -615 |
| 106635532 | SNORA100 | snoRNA | chr1 | 244854019 | 244855160 | + | 881.04 | -372 |
| 106635683 | SNORD141A | snoRNA | chr6 | 73517478 | 73519628 | + | 581.67 | -115 |
| 619567 | SNORD2 | snoRNA | chr3 | 186784087 | 186785424 | + | 322.22 | -41 |
| 6029 | RN7SL1 | scRNA | chr14 | 49586344 | 49587029 | + | 258.20 | 106 |
| 100874097 | UBE2Q1-AS1 | ncRNA | chr1 | 154552137 | 154553270 | + | 176.60 | -906 |
| 256021 | LINC01619 | ncRNA | chr12 | 92143119 | 92143635 | + | 139.33 | -546 |
| 677820 | SNORA38 | snoRNA | chr6 | 31622512 | 31623054 | + | 98.74 | -296 |
| 100616393 | MIR4657 | ncRNA | chr7 | 44881367 | 44882239 | + | 93.25 | -3 |
| 100130698 | LOC100130698 | ncRNA | chr10 | 80247218 | 80248062 | + | 91.84 | -118 |
| 407043 | MIR7-1 | ncRNA | chr9 | 83969872 | 83970474 | + | 88.17 | -316 |
| 100422885 | MIR1244-2 | ncRNA | chr12 | 12111122 | 12111734 | + | 77.81 | -524 |
| 100302233 | MIR1268A | ncRNA | chr19 | 3064733 | 3065681 | + | 72.06 | -493 |
| 100616490 | MIR4706 | ncRNA | chr14 | 65043314 | 65044123 | + | 71.66 | -970 |
| 677806 | SNORA20 | snoRNA | chr6 | 159780826 | 159781466 | + | 62.69 | -765 |
| 677811 | SNORA28 | snoRNA | chr14 | 103336446 | 103337623 | + | 61.51 | -815 |

| **Table S4. Distributions of DNA peaks binding with HIF-1α under normoxia** | | | | |
| --- | --- | --- | --- | --- |
| Annotation | Number of peaks | Total size (bp) | Log2 Ratio (obs/exp) | LogP enrichment (+values depleted) |
| 3UTR | 0 | 26833139 | -1.702 | 2.244 |
| Promoter | 4 | 35946139 | 0.418 | -1.046 |
| TTS | 1 | 32404629 | -1.432 | 1.397 |
| Exon | 1 | 37120946 | -1.628 | 1.692 |
| Intron | 111 | 1257910936 | 0.084 | -1.461 |
| 5UTR | 0 | 2601483 | -0.283 | 0.217 |
| Intergenic | 140 | 1684358172 | -0.003 | 0.67 |

| **Table S5. Annotations of non-coding RNA binding with HIF-1α under normoxia** | | | | | | |
| --- | --- | --- | --- | --- | --- | --- |
| Annotation | Number of peaks | | Total size (bp) | | Log2 Ratio (obs/exp) | LogP enrichment (+values depleted) |
| miRNA | | 0 | | 97618 | -0.012 | 0.008 |
| ncRNA | | 0 | | 7044070 | -0.667 | 0.587 |
| snoRNA | | 0 | | 357 | 0 | 0 |
| scRNA | | 0 | | 97 | 0 | 0 |
| rRNA | | 0 | | 25562 | -0.003 | 0.002 |
